# Identifying the molecular mechanisms of sepsis-associated acute kidney injury and predicting potential drugs

**DOI:** 10.3389/fgene.2022.1062293

**Published:** 2022-12-12

**Authors:** Guangfeng Guo, Yunting Wang, Wanyu Kou, Hua Gan

**Affiliations:** Department of Nephrology, The First Affiliated Hospital of Chongqing Medical University, Chongqing, China

**Keywords:** ferroptosis, immune infiltration, drug targets, septic shock-associated acute kidney injury, miRNA-mRNA net

## Abstract

**Objective:** To provide insights into the diagnosis and therapy of SA-AKI *via* ferroptosis genes.

**Methods:** Based on three datasets (GSE57065, GSE30718, and GSE53771), we used weighted co-expression network analysis to identify the key regulators of SA-AKI, its potential biological functions, and constructed miRNA‒mRNA complex regulatory relationships. We also performed machine learning and *in vitro* cell experiments to identify ferroptosis genes that are significantly related to SA-AKI in the two datasets. The CIBERSORT algorithm evaluates the degree of infiltration of 22 types of immune cell. We compared the correlation between ferroptosis and immune cells by Pearson’s correlation analysis and verified the key genes related to the immune response to reveal potential diagnostic markers. Finally, we predicted the effects of drugs and the potential therapeutic targets for septic kidney injury by pRRophetic.

**Results:** We found 264 coDEGs involving 1800 miRNA molecules that corresponded to 210 coDEGs. The miRNA‒mRNA ceRNA interaction network was constructed to obtain the top-10 hub nodes. We obtained the top-20 ferroptosis genes, 11 of which were in the intersection. We also identified a relationship between ferroptosis genes and the immune cells in the AKI dataset, which showed that neutrophils were activated and that regulatory T cells were surpassed. Finally, we identified EHT1864 and salubrinal as potential therapeutic agents.

**Conclusion:** This study demonstrated the roles of miR-650 and miR-296-3p genes in SA-AKI. Furthermore, we identified *OLFM4*, *CLU*, *RRM2*, *SLC2A3*, *CCL5*, *ADAMTS1*, and *EPHX2* as potential biomarkers. The irregular immune response mediated by neutrophils and Treg cells is involved in the development of AKI and shows a correlation with ferroptosis genes. EHT 1864 and salubrinal have potential as drug candidates in patients with septic acute kidney injury.

## Introduction

Acute kidney injury (AKI) is one of the most common and serious complications, and an independent risk factor in sepsis (Nentwich and John, 2022). Its occurrence rate is 1.9% among inpatients, but it is more common in patients with sepsis (more than 40% of sepsis intensive-care patients have AKI) (Liangos et al., 2006; Bagshaw et al., 2008; Hoste et al., 2015). Sepsis-related mortality roughly doubles when AKI occurs. Sepsis-associated acute kidney injury (SA-AKI) has become a major public health problem, leading to increased chronic kidney disease (Kellum et al., 2019; Montomoli et al., 2019). However, the pathophysiological mechanisms underlying SA-AKI are not well understood and need further exploration.

MicroRNAs (miRNAs) are a class of endogenous noncoding RNAs that consist of 19–25 nucleotides in the genomes of higher eukaryotes. They bind to target mRNA, resulting in either translational repression or degradation of the respective mRNA target (Neitemeier et al., 2017). This regulates various biological functions, including energy metabolism, proliferation, and apoptosis. These biological functions play a critical role in the progression of kidney diseases, including SA-AKI (Wei et al., 2013).

Ferroptosis is a regulated process of cell death caused by an iron-dependent accumulation of lipid hydroperoxides. Unlike apoptosis, necrosis, and autophagy, ferroptosis is characterized by cell-membrane integrity, normal nucleus size, and dense small mitochondria (Dixon et al., 2012; Xie et al., 2016; Neitemeier et al., 2017). Increasing evidence suggests that miRNAs may play a pivotal role in various renal disorders (Zhang and Li, 2022), but few studies have described the biofunction of ferroptosis genes in SA-AKI.

To identify the critical regulator of SA-AKI, we performed bioinformatic analysis by integrating three microarray datasets from the Gene Expression Omnibus (GEO) database (GSE57065, GSE30718, and GSE53771). We also constructed a ceRNA network by weighted correlation network analysis (Zhao et al., 2010; Yan et al., 2021). Furthermore, we recognized ferroptosis in AKI according to ferroptosis databases and cellular models. We had previously evaluated the degree of immune cell infiltration. We next made predictions for potential drugs and chose infiltration. Finally, we made predictions for potential drugs and chose the most significant genes as potential therapeutic targets.

## Materials and methods

### Identification of differentially expressed genes

In the present study, we downloaded three microarray datasets from the GEO database. The GSE57065 dataset is an mRNA expression dataset including 28 patients with AKI and 25 healthy volunteers. The GSE30718 dataset includes 28 patients with AKI and 11 healthy volunteers. The GSE53771 dataset is a miRNA expression dataset that includes 16 patients with AKI and 20 healthy volunteers.

We first normalized the two datasets to eliminate the difference in data amounts between different samples by using the function “normalizeBetweenArrays” from the limma R package. Then, we analyzed the difference between AKI and normal tissues in these two datasets, and the differentially expressed genes (DEGs) were those with adjusted *p* < 0.05 (all *p* values were adjusted with multiple testing) and a confidence interval of fold change greater than 95%. Finally, we took the intersection of differentially expressed genes in the two datasets as coDEGs. For miRNA datasets, we used the limma algorithm to compare AKI vs. the control and extracted differentially expressed miRNAs (demiRNA).

### Construction of the co-expression networks

All of the coDEGs that we obtained were included for constructing co-expression modules by weighted co-expression network analysis (WGCNA). According to the significance that reached adjusted *p* < 0.05, we screened out the functional gene sets that are significantly associated with AKI. These functional gene sets presented co-expression correlations at the expression level, which indicated concordance at the functional level.

### Functional enrichment analysis of co-expression modules

We used Gene Ontology (GO) terms and KEGG pathway enrichment to explore the biological functions that the functional gene sets regulated in order to analyze the mRNAs of the phenotype module using the clusterProfiler r package. The threshold for statistical significance was set at *p* < 0.05.

### Construction of a ceRNA network based on miRNA‒mRNA

To recognize the targeted regulatory relationships, we obtained miRNA‒mRNA regulatory data from three databases of miRTarBase, Tarbase, and miRecords. Only validated relationships by experimental test were included.

Based on regulatory relationships, we built the ceRNA regulatory network. Nodes in the network represent coDEGs and edges represent the targeted regulation relationship verified by experiments. The mcode plug-in of the Cytoscape software was used to perform module mining on the network and analyze the nature of the network topology.

### Extraction of hub genes

According to the analysis of the topology network, we recognized the hub genes and compared the expression and function of the hub genes *via* node degree distribution sorting.

### Analysis of ferroptosis factors

We downloaded the ferroptosis factors from the databases and previous literature (PMID: 33867820, PMID: 33767582, PMID: 33330074, and PMID: 32760210, FerreDB database). Ferroptosis factors significantly associated with acute kidney injury were identified by comparison with coDEGs. The mcode plug-in of Cytoscape was used to perform module mining on the network and analyze the nature of the network topology. Modeling and predictions were made in two datasets. The training set was used for model training and parameter optimization, and the test set was used to evaluate the performance of the model. We compared three commonly-used machine learning models: naive Bayes, support vector machines, and random forests. The ROC curve was then used to compare and evaluate the efficiency of the three models.

### Immune microenvironment analyses

The CIBORSORT algorithm was used to quantitatively analyze the immune microenvironment of each sample, including the evaluation of the degree of infiltration of 22 kinds of immune cell. Using Pearson’s correlation analysis, the correlation between the ferroptosis and each immune cell was compared, and then the key genes significantly related to the immune response were identified.

### Potential drug discovery

We used the pRRophetic package in R combined with the gene expression profile of the acute renal injury dataset to predict potential drugs. Pearson’s correlation analysis was used to identify the correlation between drugs and ferroptosis for drugs with significantly higher IC50 values in AKI samples than in normal controls.

### Cell culture and treatment

The human renal proximal tubular epithelial cell line (HK2) was provided by Dr. Jiang, Center for Kidney Disease, Chongqing Medical University. The cells were cultured in 1640 medium (Gibco, United States) containing 10% FBS (PAN-Biotech, Adenbach, Germany) and 1% penicillin‒streptomycin (Beyotime, China) at 37°C in 5% CO_2_ wet air. After the concentration of the cells reached 80%, the HK-2 cells were treated with LPS (15 μg/ml, Sigma‒Aldrich, United States) to induce kidney injury for 24 h. The cells were divided into a control group and a LPS group for further experiments.

### Statistical analysis

Data are presented as the mean ± standard deviation. GraphPad Prism 8 software (GraphPad Software, CA) and R software were used for statistical analyses. Analysis of variance or a *t*-test was used for statistical comparisons. *p* < 0.05 was considered significant.

## Results

### Differential expression analysis

**FIGURE 1:**
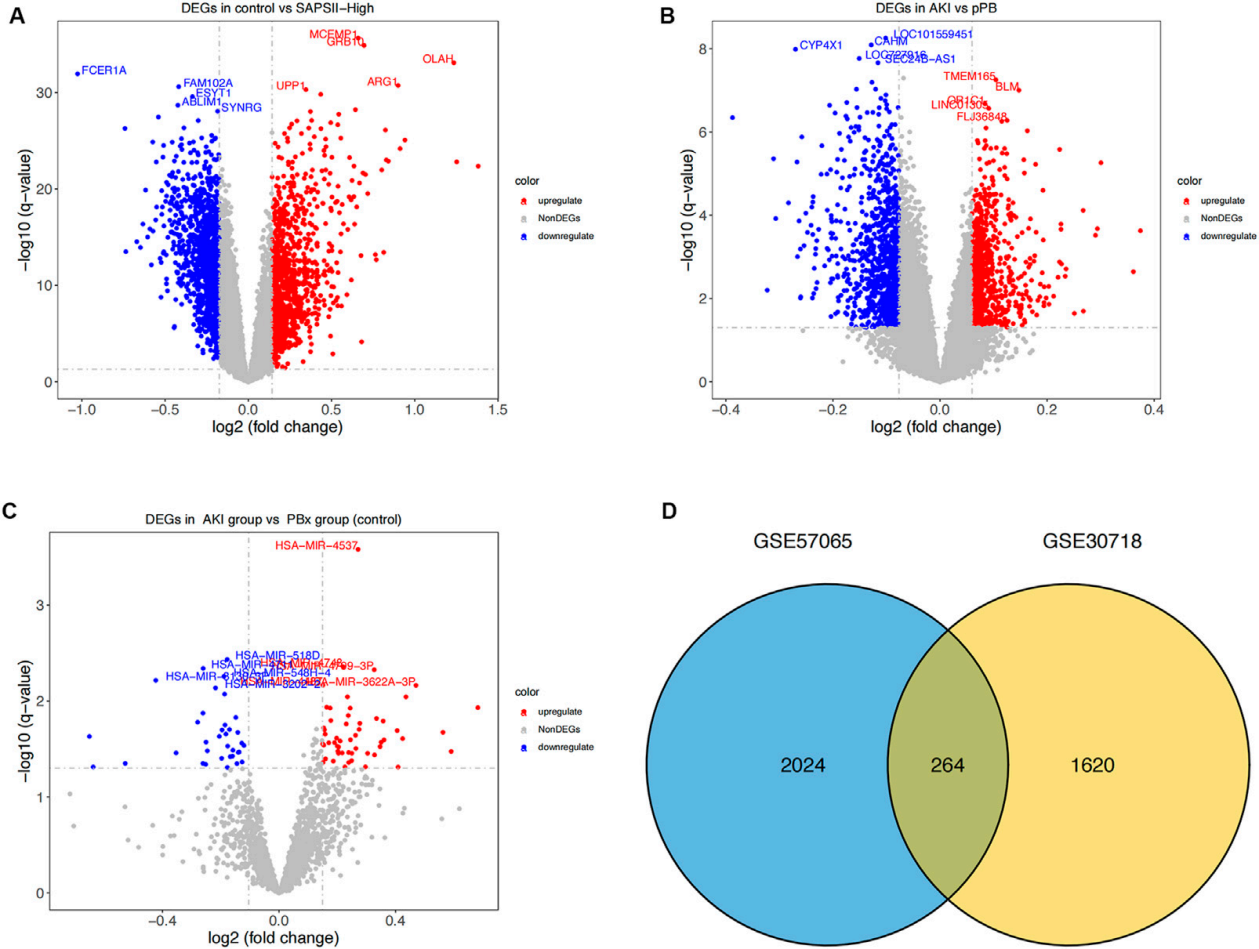
Differential expression analysis. **(A)** Distribution of differentially-expressed genes between the SAPS II-High group and the control group. *x*-axis represents the log2-fold-change; *y*-axis represents the -log10-FDR. The color scale is set up with red as upregulation and blue as downregulation. **(B)** Distribution of differentially-expressed genes between AKI and pPB. *x*-axis represents the log2-fold-change; *y*-axis represents the -log10-FDR. The color scale is set up with red as upregulation and blue as downregulation. **(C)** Distribution of differentially expressed genes between AKI and PBX. *x*-axis represents the log2-fold-change; *y*-axis represents the -log10-FDR. **(D)** Consistency of DEGs in the two datasets. Blue and yellow represent the DEGs of GSE57065 and GSE30718 respectively.

We first performed a differential analysis on the GSE57065 dataset and identified 1144 upregulated and 1144 downregulated genes (Figure 1A). We also recognized 1884 differentially expressed genes in the GSE30718 dataset, of which 899 were upregulated and 985 were downregulated (Figure 1B). From the GSE53771 dataset, 55 significantly upregulated and 35 downregulated miRNAs were found (Figure 1C). By comparing the AKI datasets of the two groups of mRNA, we screened 264 differentially expressed genes (coDEGs) (Figure 1D).

### Identification of co-expression modules for WGCNA

**FIGURE 2:**
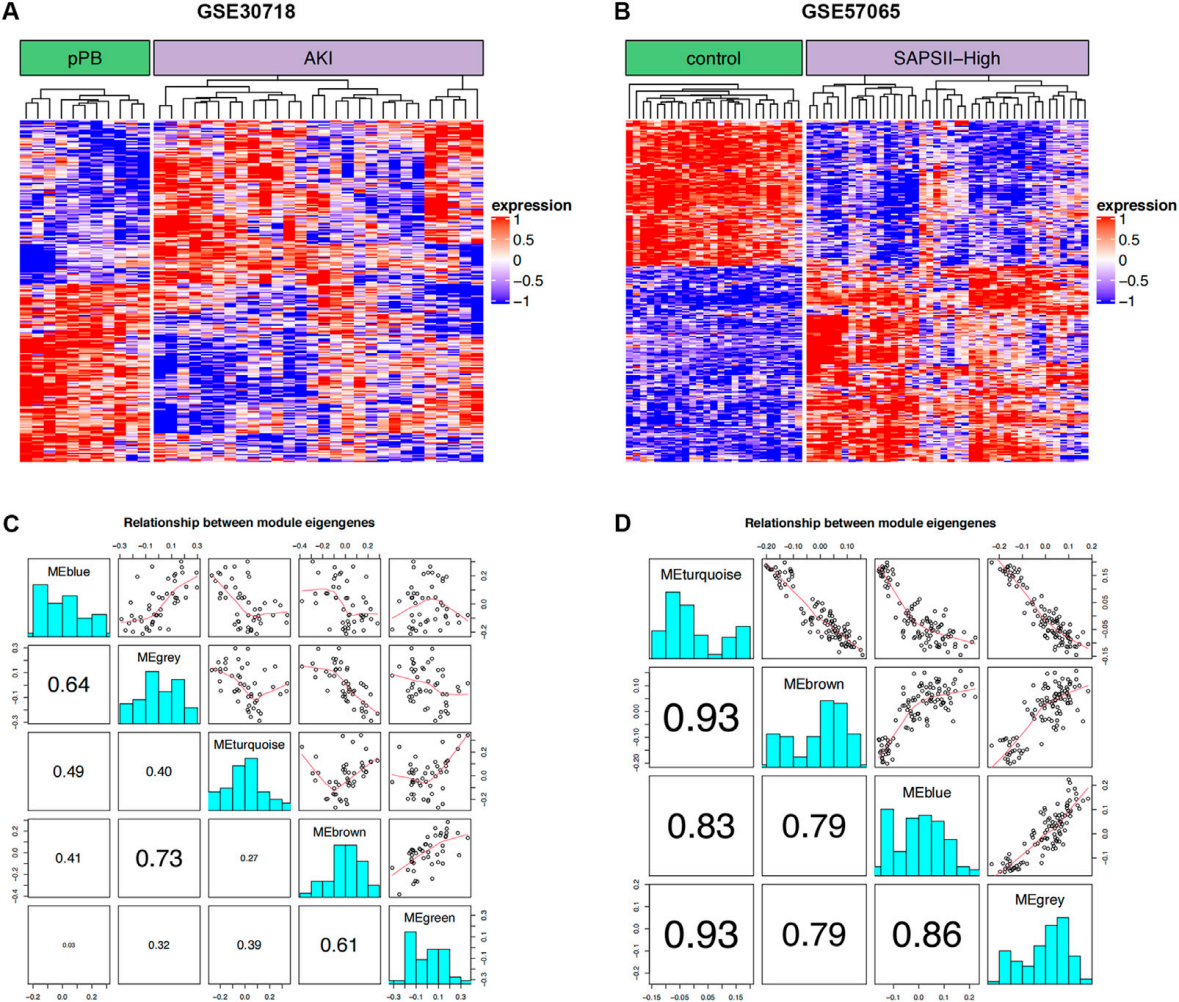
WGCNA. **(A)** Clustering of DEGs in two groups of GSE30718; red and blue represent high and low expression, respectively. **(B)** Clustering of DEGs in two groups of GSE57065; red and blue represent high and low expression, respectively. **(C)** Correlation for WGCNA modules in GSE30718. **(D)** Correlation for WGCNA modules in GSE57065.

The 264 coDEGs found in the two mRNA datasets showed a significant co-expression relationship and could significantly distinguish patients with AKI and controls or patients with septic shock (Figures 2A,B). We then used these genes to construct a weighted gene co-expression network (Figures 2C,D). The two datasets contain functional modules with a high co-expression relationship. At the same time, we identified the specificity of this correlation in patients with septic shock and those with acute renal injury. Furthermore, the co-expression relationship among the functional modules of patients with septic shock was weaker than that of acute renal injury. In contrast, the co-expression relationship was more prominent in the renal injury dataset, suggesting that these coDEGs play a significant role in the process of acute renal injury.

### Functional module enrichment analysis

**FIGURE 3:**
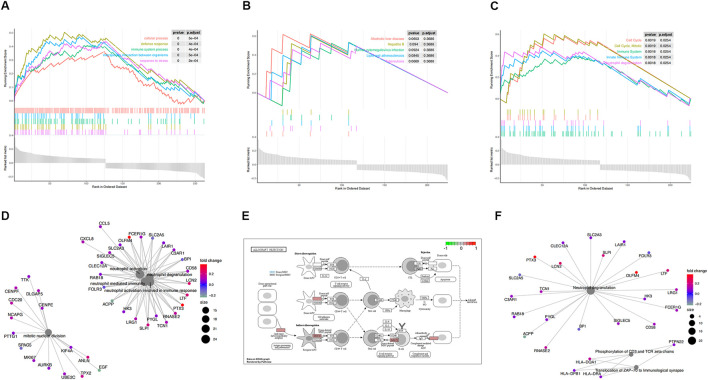
Functional enrichment analysis of GSE30718. **(A)** KEGG pathways enrichment analysis. **(B)** Gene ontology (GO) enrichment analysis. **(C)** Reactome enrichment analysis. **(D)**. emap visualizes gene ontology and gene-function relationships. **(E)** Significant enrichment pathways; green represents genes that have relatively low expression and red represents genes that have relatively high expression. **(F)** emap visualizes Reactome gene-function relationships.

**FIGURE 4:**
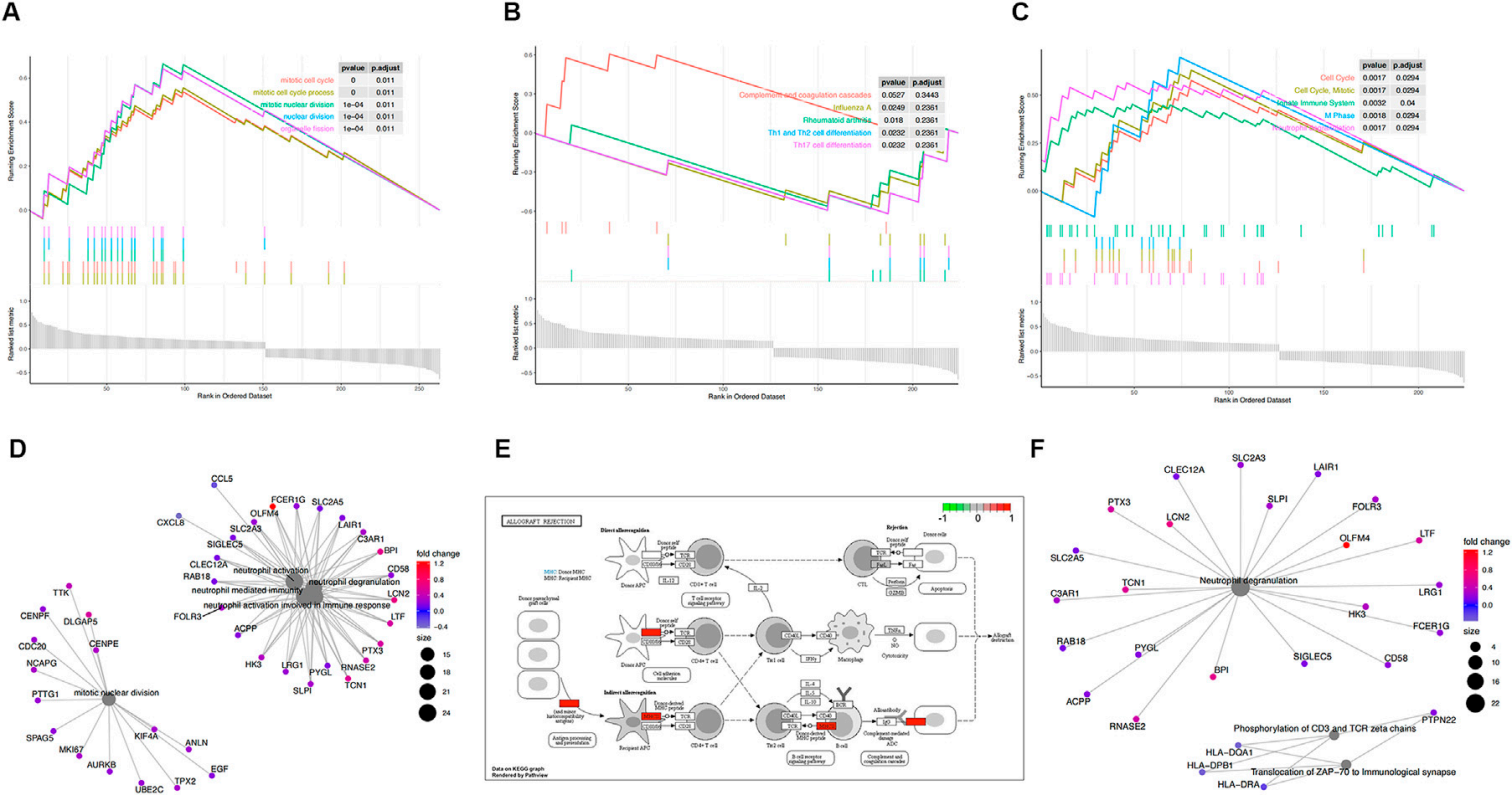
Functional enrichment analysis of GSE57065. **(A)** KEGG pathways enrichment analysis. **(B)** Gene ontology (GO) enrichment analysis. **(C)** Reactome enrichment analysis. **(D)**. emap visualizes gene ontology and gene-function relationships. **(E)** Significant enrichment pathways; green represents genes that have relatively low expression and red represents genes that have relatively high expression. **(F)** emap visualizes Reactome gene-function relationships.

To certify the biological function of the gene sets in the co-expression functional modules, we performed functional enrichment analysis for functional modules from GSE30718 (Figure 3). We also conducted a functional enrichment analysis on the functional modules from GSE57065 (Figure 4).

Parts A, B, and C in Figures 3, 4show the results of the gene set enrichment analysis (GSEA) from the GO, KEGG, and Reactome databases. We found that the coDEGs had enriched immunity and infection-related functions. This suggests that the dysfunction of the immune response induced by inflammatory infection is an important inducer of renal injury, and even septic shock, in the progression of acute renal injury.

### Investigation of miRNA–mRNA regulatory relationships

**FIGURE 5:**
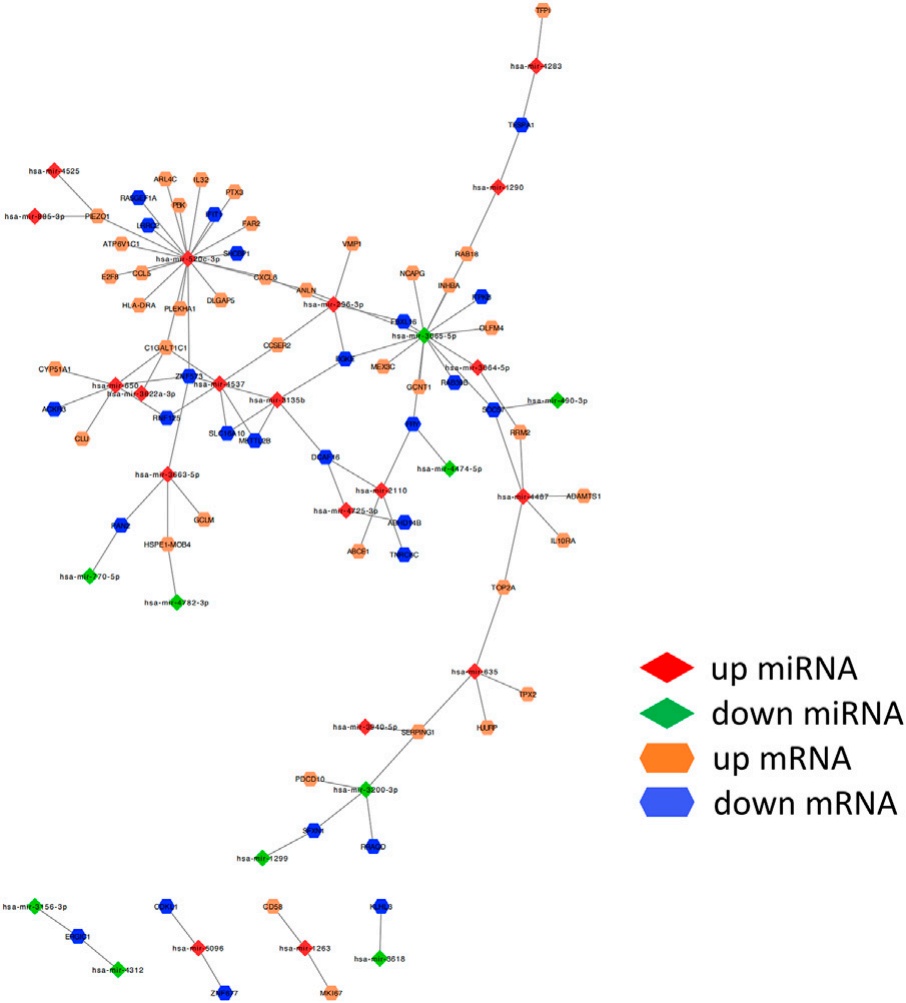
miRNA–mRNA regulatory relationships. ceRNA upregulated genes are yellow; downregulated genes are blue. The up- or down-regulated miRNA are highlighted in red and green, respectively. The edges represent miRNA–mRNA regulatory relationships.

We obtained 1800 miRNA molecules corresponding to 210 coDEGs from the miRecords, miRTarBase, and TarBase datasets. According to the 90 DEmirDNA from the GSE53771 datasets, we eventually obtained 102 regulatory relationship groups. Based on the miRNA‒mRNA regulatory relationships, the ceRNA regulatory network was built using Cytoscape software (Figure 5).

We ranked all nodes by their degree in descending order and obtained the top-10 hub nodes (Table 1). Eight nodes of these were identified, which means that these miRNAs led to abnormal function by regulating the coDEGs. Furthermore, C1galt1c1 and znf573 are regulated by four different demiRNAs. This was a comprehensive effect regulated by multiple miRNAs.

**TABLE 1:** Hub nodes in the ceRNA network.

name	Degree
hsa-mir-520c-3p	20
hsa-mir-3065-5p	12
hsa-mir-650	6
hsa-mir-296-3p	5
hsa-mir-3135b	5
hsa-mir-4487	5
hsa-mir-4537	5
C1GALT1C1	4
ZNF573	4
hsa-mir-635	4

### Analysis of ferroptosis genes

**FIGURE 6:**
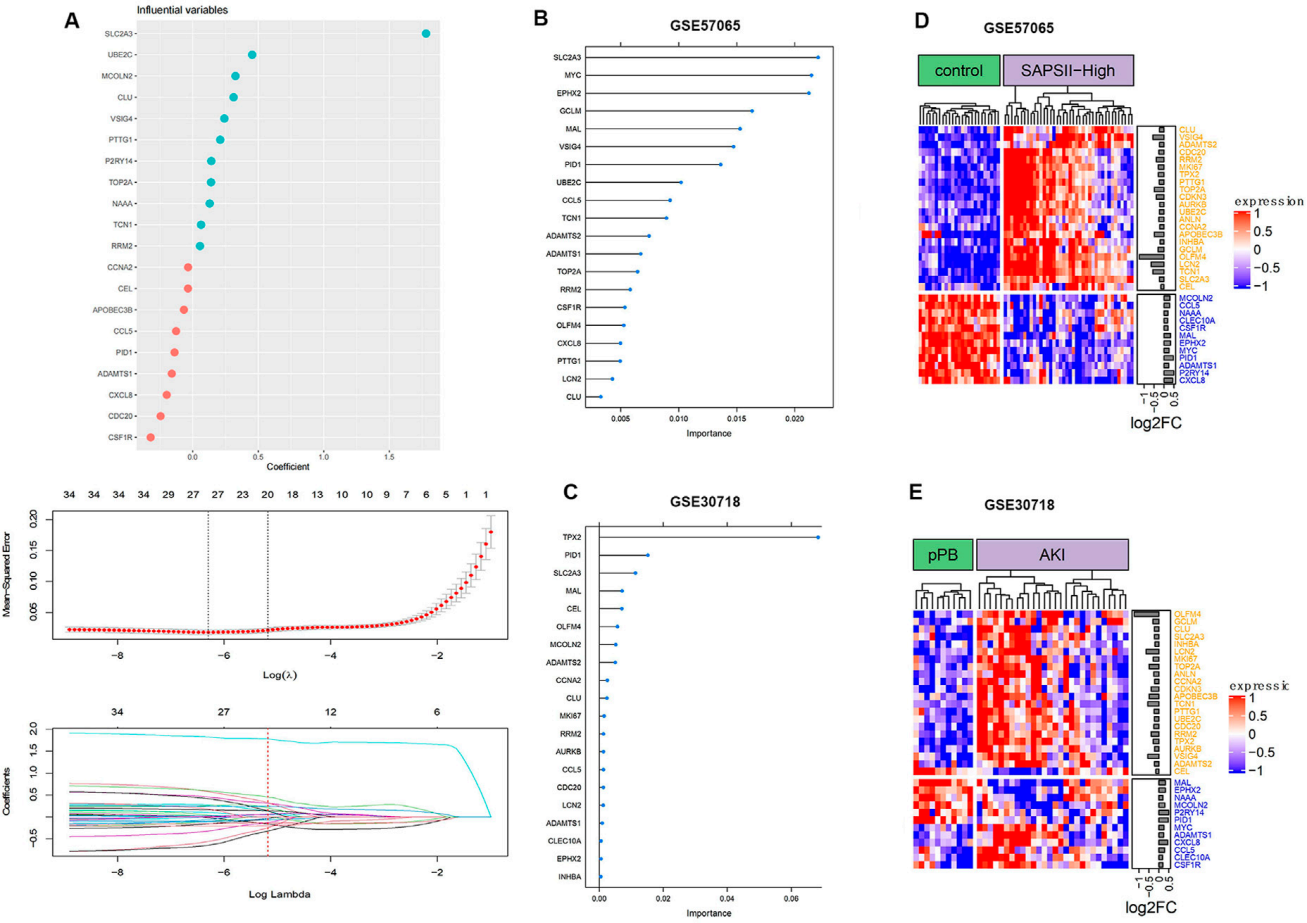
Ferroptosis gene expression distribution. **(A)** Process of feature selection in training sets; top-20 significant DEGs were screened by random forest and lasso regression analyses. **(B)** Clustering of ferroptosis genes in GSE57065. **(C)** Clustering of ferroptosis genes in GSE30718. **(D)** Importance distribution of ferroptosis signature genes in GSE57065. **(E)** Importance distribution of ferroptosis signature genes in GSE30718.

The analysis of ferroptosis factors showed that 34 genes in the 264 coDEGs were ferroptosis factors (Figure 6). We found that over 60% of genes were highly expressed in patients with AKI and septic shock. The accuracy of the three machine learning models can reach nearly 100%, indicating that the 20 characteristic genes enable a distinction between patients and controls.

### Analysis of the immune microenvironment

**FIGURE 7:**
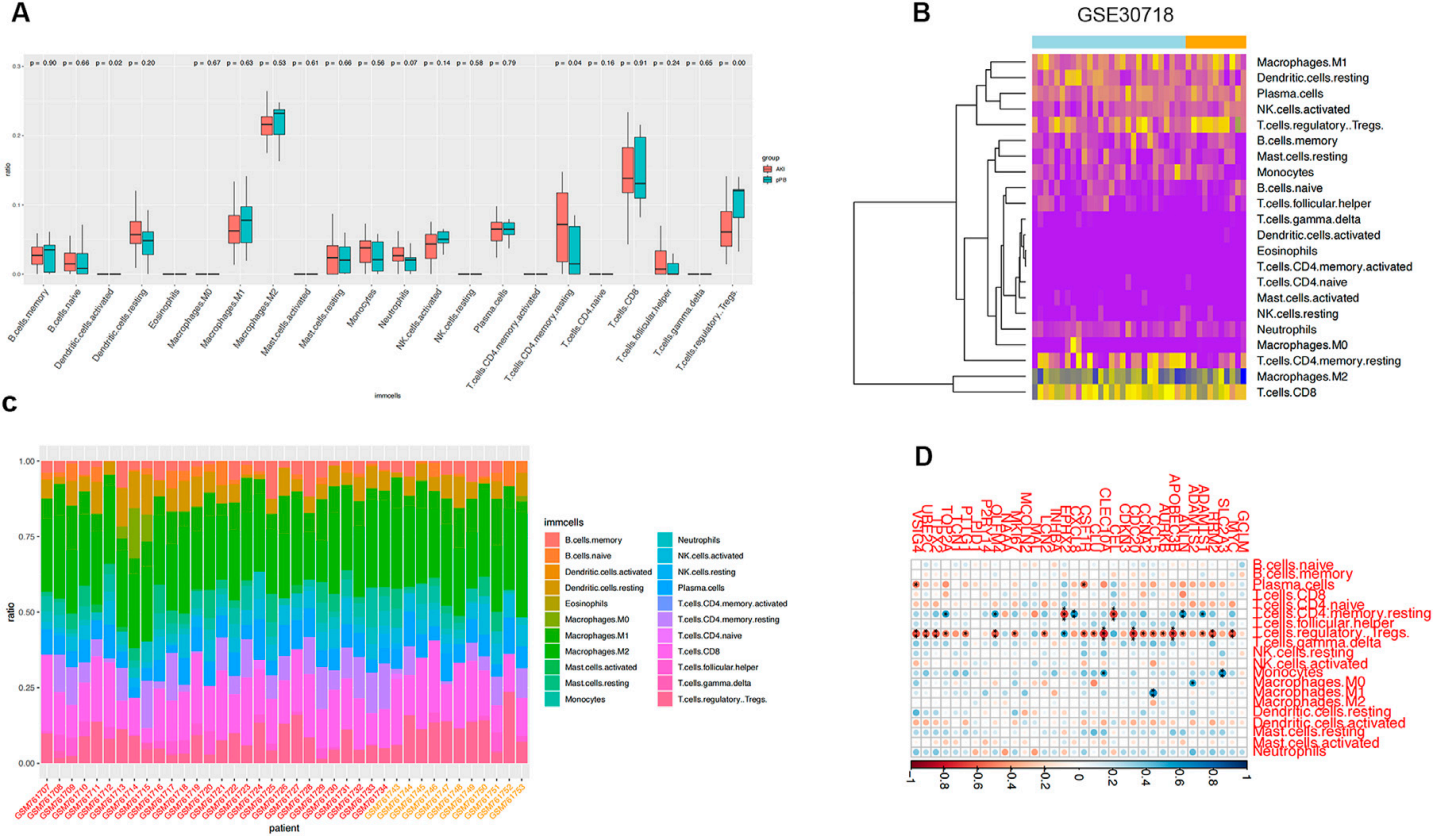
Immune infiltration analysis. **(A)** Infiltration fraction overview of 22 immune cells and comparison of infiltration scores in two groups; horizontal axis is immune cells and vertical axis is infiltration fraction. **(B)** Immune cell infiltration cluster. **(C)** Proportion of infiltrating immune cells. **(D)** Correlation analysis of ferroptosis genes and immune cells in GSE30718.

We estimated the infiltrating fractions of immune cell types in the two datasets and compared the relations between immune cell types and ferroptosis-related genes (Figure 7; Supplementary Figure S1).

We found that the expression of ferroptosis-related genes was significantly negatively correlated with Treg cells in the samples of patients with septic shock. Ferroptosis-related genes in the AKI samples were correlated with many immune cells, including T cells, B cells, and macrophages, suggesting that the dysregulated immune response participates in the development of AKI and is related to ferroptosis-related genes.

### Potential drug discovery

**FIGURE 8:**
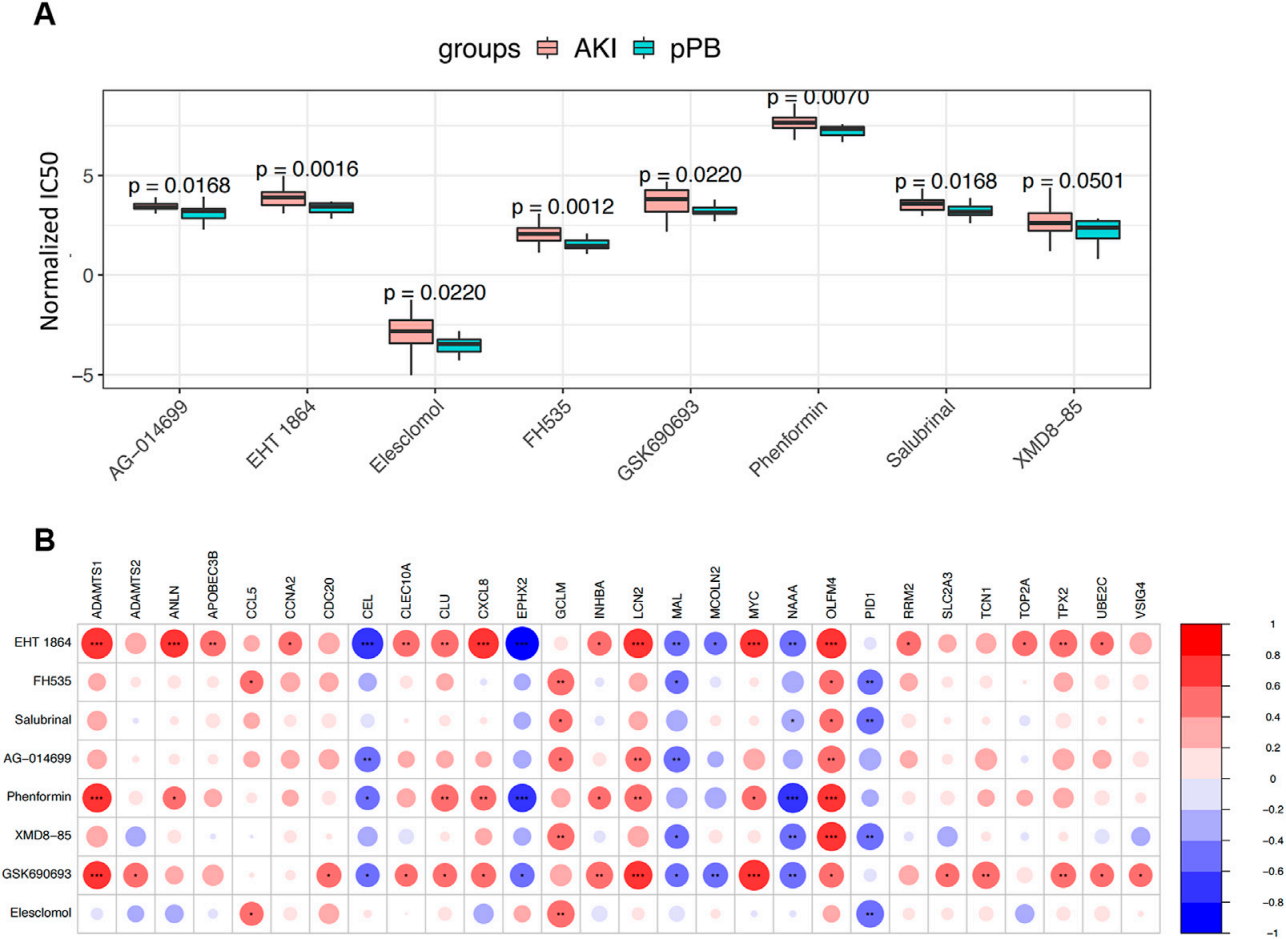
Drug Predict analysis. **(A)** Comparison of the sensitivity of screening drugs in different groups. **(B)** Screening for correlation analysis between drug sensitivity and ferroptosis genes; blue and red represent positive and negative correlations, respectively.

We estimated the potential therapeutic effects of 251 drug molecules in patients with AKI using the pRRophetic algorithm (Figure 8A). We identified eight drug molecules with significant therapeutic effects in patients with AKI; these drug molecules showed an obvious correlation with ferroptosis factors (Figure 8B). This result suggests that ferroptosis factors may be involved in the potential molecular mechanism and may become new drug targets, enabling the monitoring of treatment.

### Expression of ferroptosis-related genes in LPS-induced SA-AKI

**FIGURE 9:**
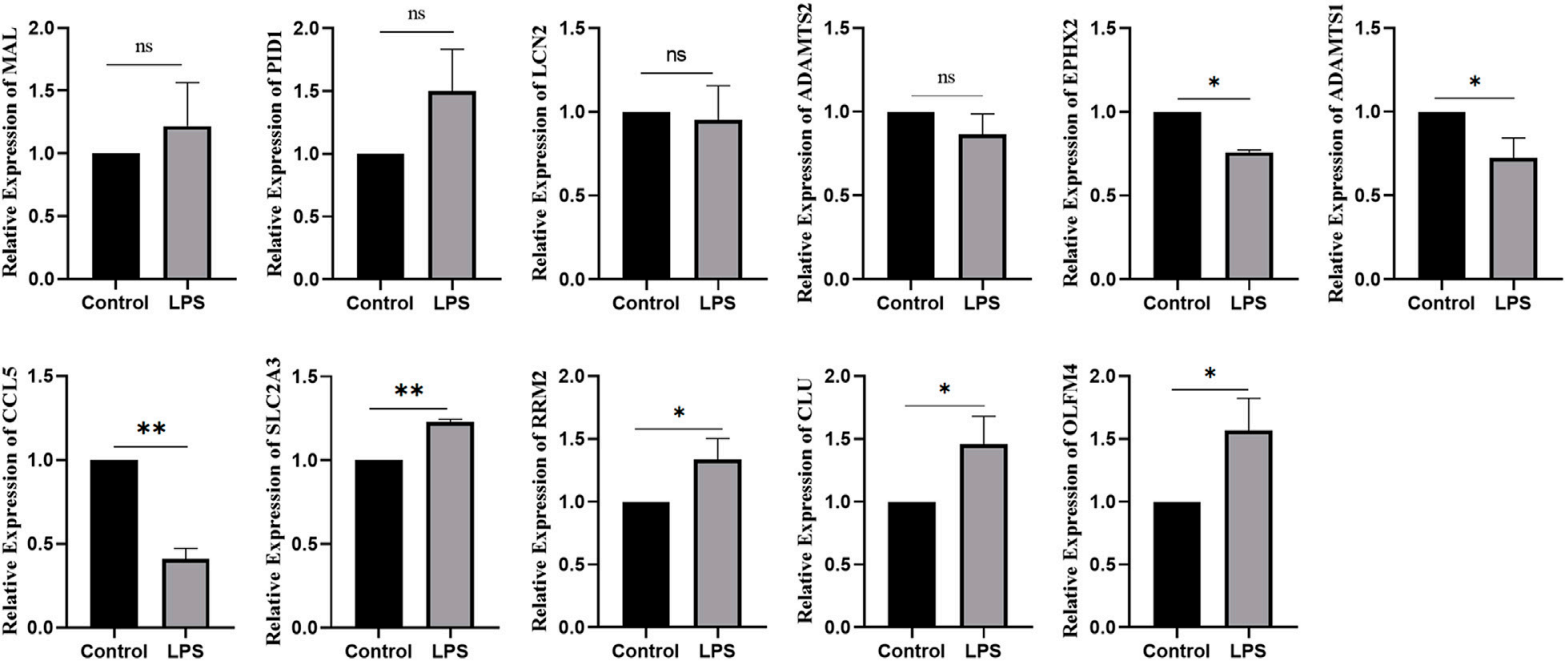
Expression of ferroptosis genes in LPS-induced SA-AKI.*:*p* < 0.05; **:*p* < 0.01.

Using bioinformatics analysis, 11 DEGs were selected as candidates. Combined with the RT‒qPCR results, the expression of *OLFM4*, *CLU*, *RRM2*, and *SLC2A3* increased in SA-AKI cell models, while the expression of *CCL5*, *ADAMTS1*, and *EPHX2* decreased in SA-AKI cell models. There was no difference in the expression of *LCN2*, *ADAMTS2*, *PID1*, or *MAL* between the two groups (Figure 9).

## Discussion

Sepsis can lead to septic shock and multiple organ dysfunction syndromes, which ultimately cause mortality. Its most common complication is AKI, which is strongly related to mortality (Basile et al., 2012; Koza, 2016). Therefore, it is critical to further elucidate the mechanism of SA-AKI using bioinformatics analysis.

The occurrence and progression of AKI are regulated by a network of multiple RNAs. WGCNA, with its ability to cluster expressed similar patterns of genes and analyze associations between modules and specific traits or phenotypes, is an analytical approach widely used in the search for the hub genes of diseases (Kwon et al., 2019; Qin et al., 2020). It is used to study human diseases to screen biomarkers and to elucidate the molecular mechanism behind the development of a disease (Yin et al., 2018; Shang et al., 2019). In our study, the co-expression relationship of the functional modules in AKI was closer than that in septic shock, suggesting that the coDEGs we found played a key role in AKI.

The etiology of AKI is variable and its pathogenesis is complicated. miRNA profiles can provide valuable interpretations for pathogenesis studies. Moreover, the high specificity and sensitivity of miRNAs in plasma and urine make miRNAs promising biomarkers for monitoring the disease process of AKI (Lee et al., 2018). In this study, we found that miR-520c-3p, hsa-mir-3065-5p, hsa-mir-650, hsa-mir-296-3p, hsa-mir-3135b, hsa-mir-4487, hsa-mir-45, and hsa-mir-635 could regulate more coDEGs, thereby leading to diastolic function. In addition, *C1GALT1C1* and *ZNF573* are regulated by four different demiRNAs, which means that their expression is the outcome of composite regulation by multiple miRNAs.

Jin et al. (2017) revealed that an increasing level of miR-650 was associated with the downregulation of B-cell CLL/lymphoma 11B (BCL11B) gene expression in acute renal allograft rejection. The upregulation of miR-650 significantly suppressed cell proliferation and induced apoptosis, which influenced AKI. Mitochondria play an important role in AKI by orchestrating cellular energy transformation, apoptosis, and reactive oxygen species (ROS) signaling. miR-296-3p can induce mitochondrial abnormalities in structure and function, which impair the proliferative potential as well as the ability to repair tubular cells (Farahani et al., 2020).

MiR-296-3p increases the expression of Bax/Bcl-2 and cleaved caspase 3/caspase 3 in SV40-MES13 mouse mesangial cells (Tang et al., 2020). We speculate that the upregulation of miR-296-3p may mediate SA-AKI by affecting mitochondrial functions or inducing apoptosis. These findings suggest that miR-650 and miR-296-3p may be potential therapeutic targets for SA-AKI. The mechanism of other miRNAs in AKI remains incompletely understood—we need to further explore the correlation between miRNAs and SA-AKI.

Ferroptosis is an iron-dependent form of nonapoptotic cell death characterized by the accumulation of intracellular lipid reactive oxygen species; it is driven by the loss of activity of the lipid repair enzyme glutathione peroxidase 4 (GPX4) (Doll et al., 2019). In this study, we found that 34 ferroptosis-related genes were differentially expressed in SA-AKI. We also used machine learning to identify the top-20 ferroptosis genes significantly related to SA-AKI. Finally, 11 ferroptosis genes were obtained after the intersection. *OLFM4*, *LCN2*, CLU, *RRM2*, *ADAMTS2*, and *SLC2A3* were members of the crucial upregulated gene set, and the crucial downregulated gene set included *PID1*, *MAL*, *CCL5*, *ADAMTS1*, and *EPHX2*. Further experiments, such as qRT‒PCR, were conducted to further confirm our findings. In our research, *OLFM4*, *CLU*, *RRM2*, and *SLC2A3* were highly expressed in LPS-induced AKI models, and *CCL5*, *ADAMTS1*, and *EPHX2* were expressed at low levels in LPS-induced AKI models. The differences were significant.

It was previously shown that *OLFM4* can negatively regulate the defense response against bacterial infections (Liu et al., 2012). It can promote leukocyte-mediated migration, neutrophil activation and degranulation (Banerjee et al., 2017). Junyu Lu et al. (2020) reported that *OLFM4* was identified as the key gene in sepsis due to its upregulated expression, suggesting that it is closely associated with sepsis. Julie E. Stark et al. (2020) found that the increase in *OLFM4* is closely related to SA-AKI and that decreased kidney function is a possible contributing factor for *OLFM4*-mediated mortality in sepsis. *CLU*, an extracellular chaperone, is a multifunctional secreted glycoprotein involved in cell adhesion, tissue remodeling, cell cycle control, apoptosis initiation, and DNA repair (Sun et al., 2020). Previous studies demonstrated the increased expression of *CLU* in early-phase AKI, unilateral ureteral obstruction, and nephrectomy (Weng et al., 2021). This has also been demonstrated in many clinical studies (Fuchs and Hewitt, 2011). Urine *CLU* has been extensively studied as a biomarker in AKI; it is superior to traditional indices in the diagnosis of proximal renal tubular injury. It not only shows high diagnostic value in severe acute kidney injury caused by high-dose drugs but has also been identified in some animal experiments. We also verified that *CLU* was increased in patients with SA-AKI and that it is likely to be a new biomarker for recognizing SA-AKI. *RRM2* encodes one of the small subunits of ribonucleotide reductase (RR), the rate-limiting enzyme for the production and repair of deoxyribonucleotides. The overexpression of *RRM2* significantly enhances the invasiveness and metastasis of cancer cells and plays a crucial role in cell proliferation and apoptosis, making it an important anticancer therapeutic target (Aye et al., 2015; Chen et al., 2019; Xiong et al., 2021). Osako Y et al. (2019) reported that a higher expression of *RRM2* is associated with carcinogenesis in clear-cell renal cell carcinoma cells. Furthermore, iron metabolism plays a crucial role in ferroptosis. That *RRM2* is critical for SA-AKI is a reasonable assumption because it is an iron metabolism-related gene and ferroptosis factor, providing a theoretical basis for further research on biomarkers. *SLC2A3* encodes the predominantly neuronal glucose transporter 3 (*GLUT3*), which is involved in energy metabolism (Ziegler et al., 2020). The study showed that *SLC2A3* was highly expressed in kidney tissue from the IRI group compared with the sham group. Furthermore, knockdown of *SLC2A3* significantly rescued decreased cell viability, increased iron levels, and increased ROS caused by ferroptosis (Wei et al., 2022). These results revealed the role of *SLC2A3* in promoting ferroptosis. The study showed that *SLC2A3* was highly expressed in kidney tissue from the IRI group compared with the sham group. Furthermore, the knockdown of *SLC2A3* significantly rescued the decreased cell viability, increased iron levels, and increased ROS levels caused by ferroptosis. These results revealed the role of *SLC2A3* in promoting ferroptosis. However, no study reported *SLC2A3* as having a significant role in SA-AKI. In our study, RT‒PCR demonstrated that the expression of *SLC2A3* was significantly upregulated in SA-AKI induced by LPS compared with controls, which accords with the bioinformatics analysis results.

*CCL5*, also known as RANTES, belongs to the family of chemotactic chemokines that promotes leukocyte trafficking to the site of infection and stimulate cytokine production (Patterson et al., 2021). Jaclyn R. Daniels et al. (2021) found that serum *CCL5* levels in AKI requiring dialysis were associated with renal recovery. *CCL5* targets monocytes, macrophages, lymphocytes, and endothelial cells during healing *via* receptors on immune cells (Balaji et al., 2015). *EPXH2* encodes soluble epoxide hydrolase (sEH). A recent study in Ephx2 knockout mice showed that the loss of epoxide hydrolase activity and phosphatase activity worsened kidney ischaemia–reperfusion injury (Zhu et al., 2016), which is contrary to the outcome in mice with kidney ischaemia-reperfusion injury that received sEH inhibitors (Shen and Hammock, 2012). The expression of *Ephx2* is decreased in SA-AKI. Further studies are needed to clarify whether the Ephx2 gene plays a protective role in AKI. *ADAMTS1* protects the kidney by limiting pericyte detachment after injury, promoting vascular stability and reducing interstitial fibrosis. Meanwhile, *ADAMTS1* has been identified as a marker of pericyte activation (Schrimpf et al., 2012). Further studies are needed to determine whether *ADAMTS1* is a potential biomarker in renal injury.

In our study, gene set enrichment analysis (GSEA) indicated that the DEGs were enriched for immune system processes, including the innate immune system and neutrophil degranulation. We performed an immune infiltration analysis and found that neutrophils were overexpressed in AKI. Neutrophils is the key effector cell of the innate immune system and plays a critical role in promoting antimicrobial functions. The imbalance of inflammatory cells, especially the increase in neutrophils and decrease in lymphocytes, participates in the pathological process of AKI. Excessive stimulation of neutrophils leads to damage of vascular permeability and endothelial function, and plays an important role in the occurrence of AKI (Akcay et al., 2009). Tregs, a special T-cell subtype, have immunosuppressive and anti-inflammatory properties (Akcay et al., 2009). In the present study, we observed that Tregs were downregulated and negatively associated with many ferroptosis genes in AKI. The mechanism of renoprotective effects in Tregs is the inhibition of the innate immune response to renal injury (Kinsey et al., 2009). In addition, Tregs can be transported to inflammatory areas to reduce the immune response (Kinsey et al., 2009). Thus, the infusion of Tregs or their pharmacological recruitment protect against ischaemic AKI.

We used DEGs in this study of CMap analysis for drug discovery and found that Eht1864 and salubrinal are potential therapeutic agents for the prevention and treatment of sepsis-induced AKI. No study has investigated the effects of Fh535, ag-014699, phenformin, xmd8-85, gsk690693, and elesclomol on anti-inflammation. On the other hand, further research on these drugs may lead to new treatments. HT1864 is a specific RAC inhibitor. RAC 1/2 are essential components of the reactive oxygen species (ROS)-generating NADP(H) oxidase system. The rise in oxidative stress leads activated NF-κB to promote proinflammatory responses, leading to renal damage (Wang et al., 2015). Samik Patel et al. (2019) found that EHT 1864 could block TGF-β1–induced fibrotic reprogramming in kidney epithelial cells and fibroblasts. The renal protection of EHT 1864—partly by inhibiting inflammatory signals in several models of chronic kidney disease—has been previously reported (Shibata et al., 2008; Kawarazaki et al., 2012; Nagase et al., 2016). Endoplasmic reticulum stress (ERS) is an early stress event of cell injury that mainly affects inflammatory response and cell death. The upregulation of key UPR markers in various renal diseases indicates that ESR plays an important role in renal injury (Cybulsky, 2017). Some studies have shown that inhibiting ER stress can reduce acute injury and prevent the transition from AKI to CKD. In an H9c2 cardiomyocyte sepsis model, salubrinal reduces mitochondrial damage by inhibiting endoplasmic reticulum stress, thereby reducing the rate of cardiomyocyte apoptosis (Jiang et al., 2022). Furthermore, salubrinal is a potential therapeutic agent for inflammatory diseases with the ability to mediate the NF-κB signaling pathway for the elimination of infiltrated inflammatory cells (Doll et al., 2019). Therefore, we can assume that salubrinal may contribute to the remission of septic AKI.

However, our study has some limitations. It is necessary to perform further experimental verification *in vivo* and *in vitro* to clarify the important role of related genes.

## Conclusion

This study provides new insights into the contribution of SA-AKI pathogenesis and strengthens the potential objectives of further research. We performed bioinformatics analysis to search for the hub genes involved in the pathogenesis of septic acute renal injury and then demonstrated the important role of miR-650 and miR-296-3p. Combined with *in vitro* cell experiments, we also identified *OLFM4*, *CLU*, *RRM2*, *SLC2A3*, *CCL5*, *ADAMTS1*, and *EPHX2* as potential biomarkers of AKI. Our study identified several new molecular drugs as potential therapeutic candidates for patients with SA-AKI; these require further research to verify whether they can be used in clinical treatment.

## Data Availability

The datasets presented in this study can be found in online repositories. The names of the repository/repositories and accession number(s) can be found in the article/Supplementary Material.

## Supplementary material

Supplementary Figure S1 Immune infiltration analysis. **(A)** Infiltration fraction overview of 22 immune cells and a comparison of infiltration scores in two groups. The horizontal axis is immune cells and the vertical axis is infiltration fraction. **(B)** Immune cell infiltration cluster. **(C)** The proportion of the infiltrating immune cells. **(D)** Correlation analysis of ferroptosis genes and immune cells in GSE57065.
