# Supplementary figures and images for: Identifying the molecular mechanisms of sepsis-associated acute kidney injury and predicting potential drugs
Source: Front Genet. 2022 Dec 12;13:1062293. doi: 10.3389/fgene.2022.1062293 (PMC9792148; doi:10.3389/fgene.2022.1062293)

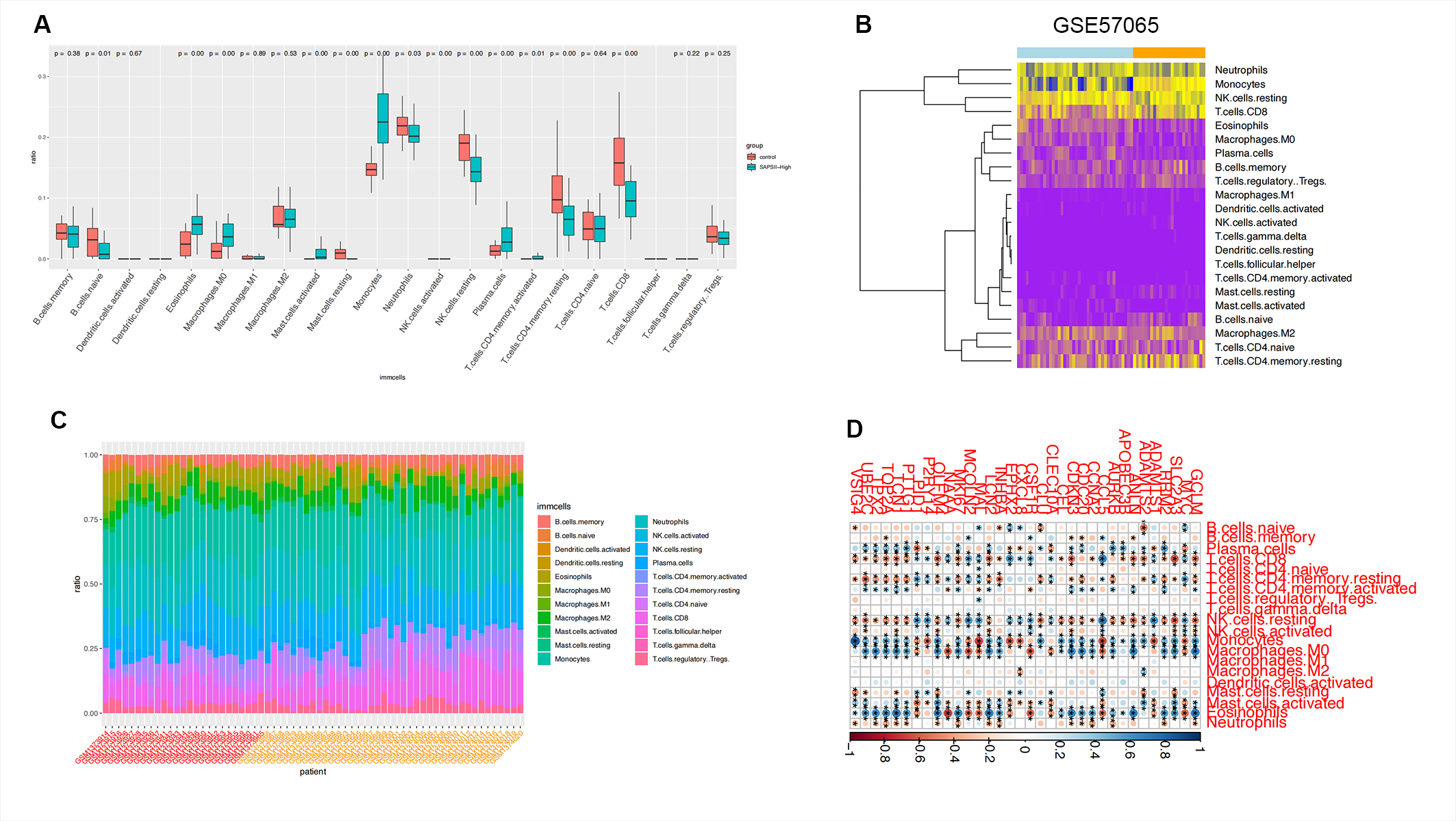

Supplement: Supplementary file 1 [file Image1.TIF]
